# Cataract and optic disk drusen in a patient with glycogenosis and di George syndrome: clinical and molecular report

**DOI:** 10.1186/s12886-017-0499-y

**Published:** 2017-06-28

**Authors:** D. Allegrini, S. Penco, A. Pece, A. Autelitano, G. Montesano, S. Paci, C. Montanari, A. Maver, B. Peterlin, G. Damante, L. Rossetti

**Affiliations:** 1 Eye Unit, Humanitas Gavazzeni Hospital, Humanitas University, Bergamo, Italy; 2 Medical Genetics Unit, Niguarda Ca’ Granda Hospital, Milan, Italy; 3 Eye Unit, Melegnano Hospital, Vizzolo Predabissi, Milan, Italy; 4 Eye Unit, San Paolo Hospital, University of Milan, Milan, Italy; 5 Pediatric Department, San Paolo Hospital, University of Milan, Milan, Italy; 6 Clinical Institute for Medical Genetics, University Medical Center Ljubljana, Ljubljana, Slovenia; 7 Medical Genetics Unit, University of Udine, Udine, Italy

**Keywords:** Glycogen storage disease type IA, Hypoglycemic cataract, Hypocalcemic cataract, DiGeorge syndrome, Optic disk drusen, Microdeletion 22q11.2

## Abstract

**Background:**

We report the ophthalmic findings of a patient with type Ia glycogen storage disease (GSD Ia), DiGeorge syndrome (DGS), cataract and optic nerve head drusen (ONHD).

**Case presentation:**

A 26-year-old white woman, born at term by natural delivery presented with a post-natal diagnosis of GSD Ia. Genetic testing by array-comparative genomic hybridization (CGH) for DGS was required because of her low levels of serum calcium. The patient has been followed from birth, attending the day-hospital every six months at the San Paolo Hospital, Milan, outpatient clinic for metabolic diseases and previously at another eye center. During the last day-hospital visit, a complete eye examination showed ONHD and cataract in both eyes. Next Generation Sequencing (NGS) was subsequently done to check for any association between the eye problems and metabolic aspects.

**Conclusions:**

This is the first description of ocular changes in a patient with GSD Ia and DGS. Mutations explaining GSD Ia and DGS were found but no specific causative mutation for cataract and ONHD. The metabolic etiology of her lens changes is known, whereas the pathogenesis of ONHD is not clear. Although the presence of cataract and ONHD could be a coincidence; the case reported could suggest that hypocalcemia due to DGS could be the common biochemical pathway.

## Background

We report the clinical and molecular details of a complex patient presenting cataract and optic disk drusen with glycogen storage disease (GSD) type Ia and DiGeorge syndrome (DGS). Type Ia GSD is due to glucose-6-phosphatase (G6P) deficiency and presents either at birth with enlarged liver or, more frequently, at the age of 3–4 months with symptoms of hypoglycemia induced by fasting (tremors, convulsions, cyanosis and apnea) [1].

DGS is due to a microdeletion in chromosome 22q11.2, whose common signs include heart disease, palate anomalies, dysmorphic facial features, developmental delay, immune deficiency and hypocalcemia associated with hypoparathyroidism.

## Case presentation

This case was studied at the San Paolo Hospital, Milan, Italy, in full respect of the Declaration of Helsinki and national laws for the protection of personal data; the patient signed an informed consent form. She is being followed in the Pediatric Department.

The patient was a 26-year-old Caucasian white woman, born at term by natural delivery. As a newborn she had had recurrent episodes of vomiting, and at four months she was diagnosed with GSD Ia, by liver biopsy. Genetic testing for G6PC gene (NM_000151.3) indicated as causative mutations c.551G > T (p.Gly184Val) and c.809G > T (p.Gly270Val) responsible for GSD Ia (OMIM:232,200).

**Fig. 1:**
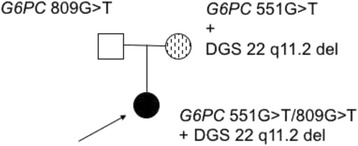
Pedigree of the complex case. The index affected case is indicated by the *arrow*. Squares represent males; *circles*, females. Genetic status of people subjected to genetic test is shown above the corresponding symbol

Over the years, on account of persistent hypocalcemia, genetic testing for DGS was requested; array-comparative genomic hybridization (CGH) confirmed the presence of a 2.42 Mb deletion from nucleotide 19.023.824 to 21.44.0514 of the q11.2 region of chromosome 22. Further analysis indicated that the deletion was inherited from the mother who also presented hypocalcemia, but none of the typical ocular signs of DGS. Psychiatric and psychological evaluations indicated that the patient has an intellectual disability (Fig. 1).

The patient has been followed from birth, with day-hospital check-ups every six months at the San Paolo Hospital, Milan, outpatient clinic for metabolic diseases, pediatric department. Currently she is under strict dietary control. Her clinical features related to DGS and GSD Ia are summarized in Table 1. She had previously also been followed at another eye center which, however, retains no imaging documentation. The eye check in our clinic was requested by the day hospital at the hospital’s center for metabolic diseases.

**Table 1:** Clinical features of the patient related with DiGeorge syndrome and Glycogen Storage Disease Ia

	SYSTEMIC CHANGES	OCULAR CHANGES
GSD IA	hepatomegaly, short stature, hypotonia	peripheral punctate opacities of the lens
DGS	cleft lip and palate, dental enamel hypoplasia, hypoparathyroidism, intellectual disability	subcapsular cataract, retinal vascular tortuosity, eyelid hooding, strabismus, and astigmatism

The patient had Best Corrected Visual Acuity (BCVA) of 20/25 (+1.00sfere, −2.00 cylinder/60°) in right eye (OD) and 20/500 (−1.00cylinder/110°) in left eye (OS). Orthoptic examination found exotropia of 25 prismatic diopters (PD) in OS, not alternating, with dominance in OD. The OS was amblyopic, because the patient had previously refused the use of glasses and occlusion in OD. Pupils were equal, round and reactive to light, without restriction or overaction. She had a clear cornea, deep, quiet anterior chambers and eyelid hooding in both eyes (OU). Applanation tonometry measured 15 mmHg OU.

**Fig. 2:**
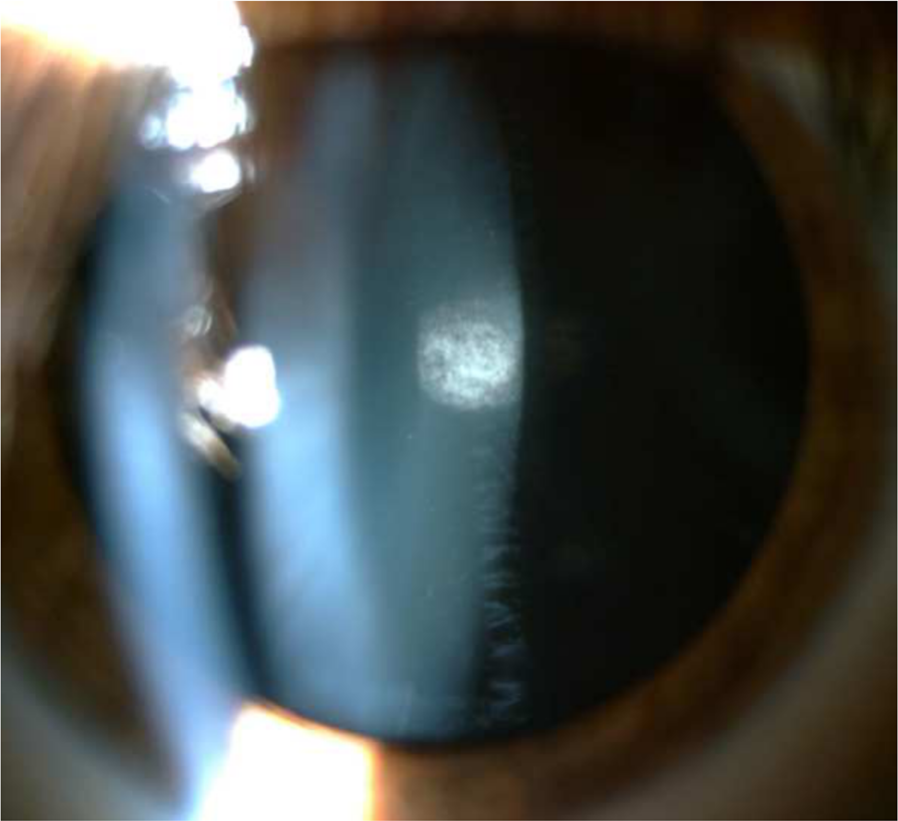
Posterior subcapsular cataract in left eye

**Fig. 3:**
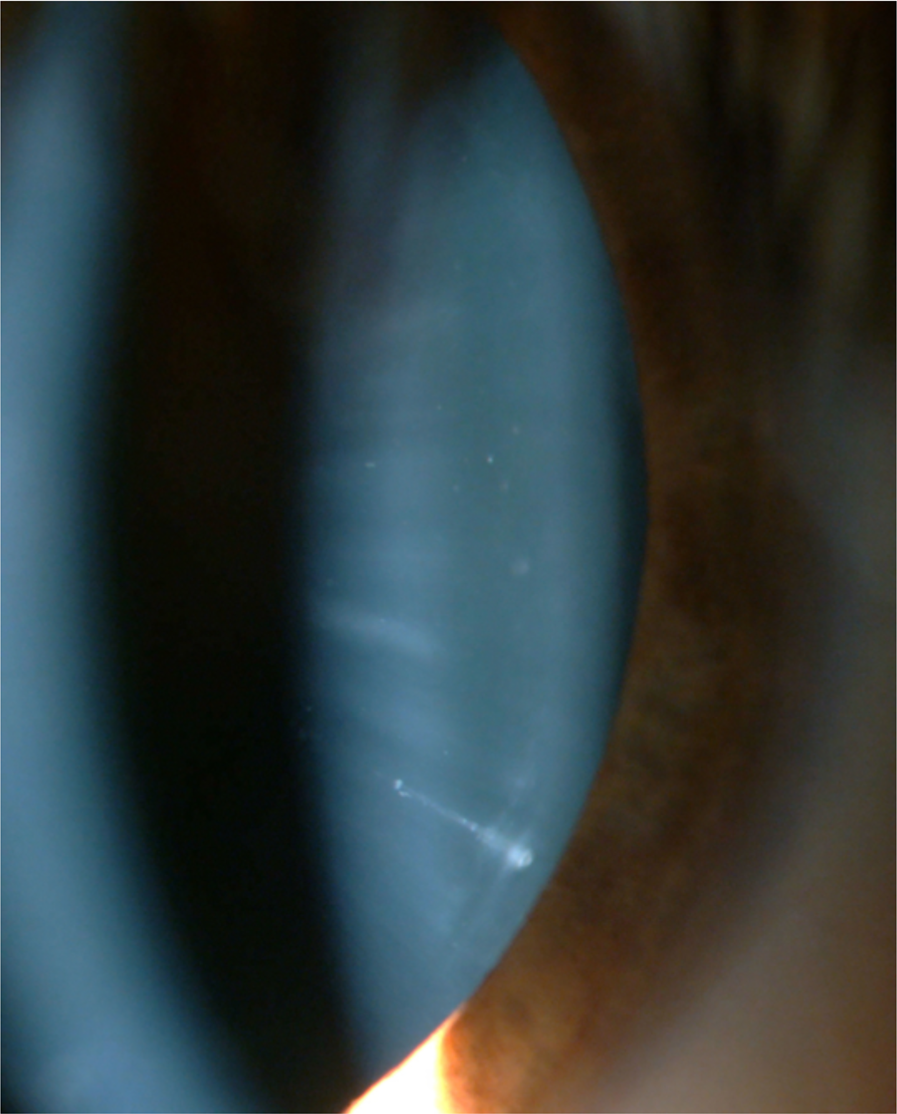
Peripheral opacities of the lens in left eye

**Fig. 4:**
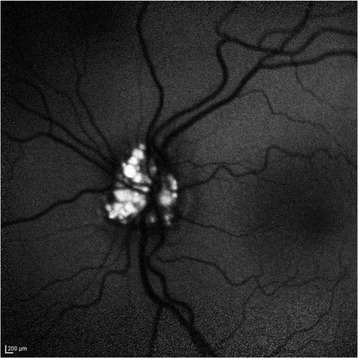
Strong autofluorescence of drusen of optic disk in left eye

**Fig. 5:**
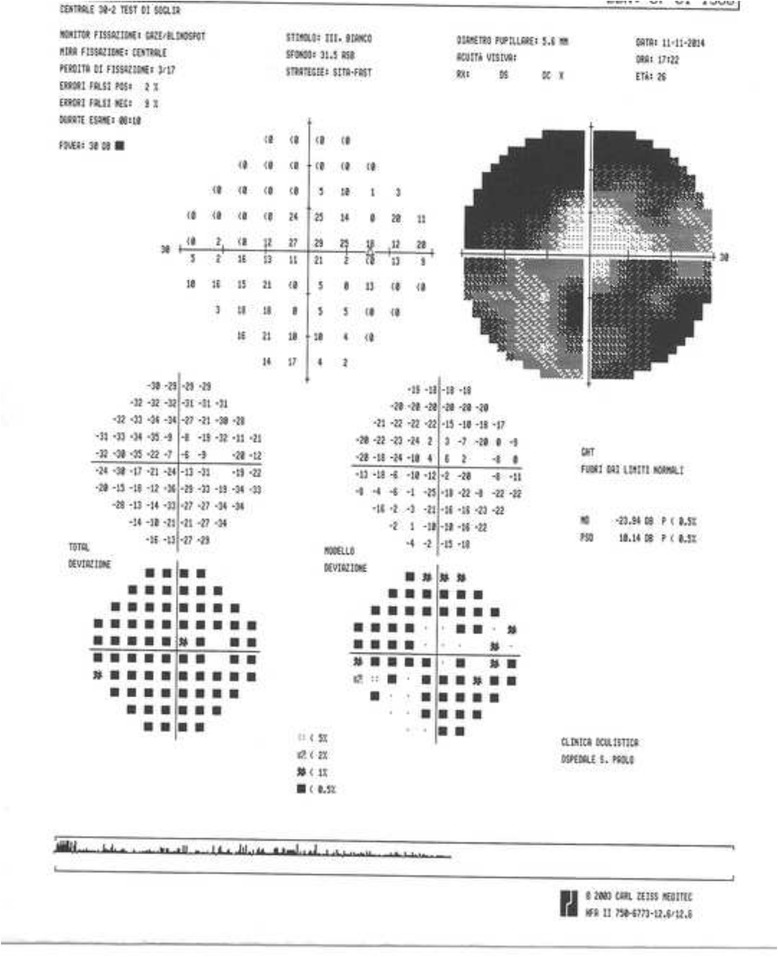
Computerized visual field: limited area of central sensitivity in right eye

**Fig. 6:**
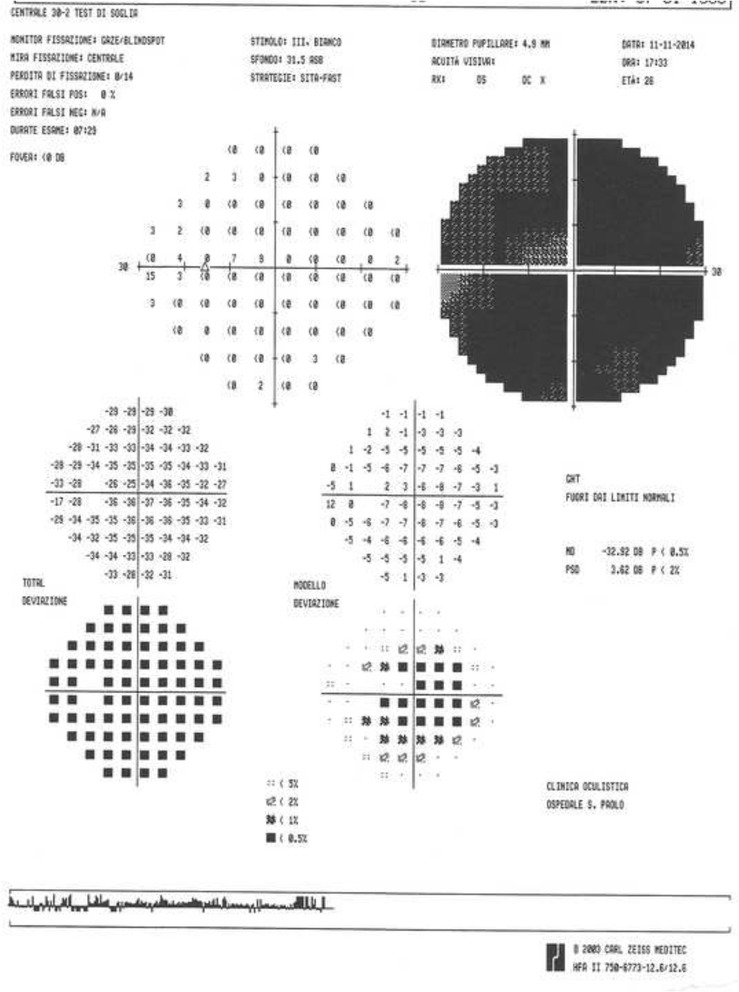
Computerized visual field: widespread loss of sensitivity in left eye

In the anterior segment there was: a) a posterior subcapsular cataract (Fig. 2), which it was manifested at the age of 18 years in OS more than in OD and it was gradually increased over the years; b) numerous peripheral punctate opacities of the lens on 360° (Fig. 3) in OU, not previously described. We strongly recommended surgical cataract extraction in OS but the patient refused surgery. The fundus presented a clear macula, tortuous vessels and numerous bright yellow ONHD, clearly visible in B scan ultrasound and autofluorescence HRA-II (Heidelberg Engineering, Heidelberg, Germany) (Fig. 4). ONHD were manifested first in OS at age 11 and then in OD at age 13, increasing in number and size over the years. A computerized visual field examination (Humphrey perimetry) at the day hospital showed advanced constriction of the OS greater than OD. It also helped explain the central vision loss in OU caused by the fixation splitting defect (Figs. 5, 6) based on the advanced visual field loss in each eye. We believed that the drusen were the main cause of the widespread reduction of the visual field; in fact the lens opacities were not diffuse, but centrally subcapsular and peripherally punctate.

**Fig. 7:**
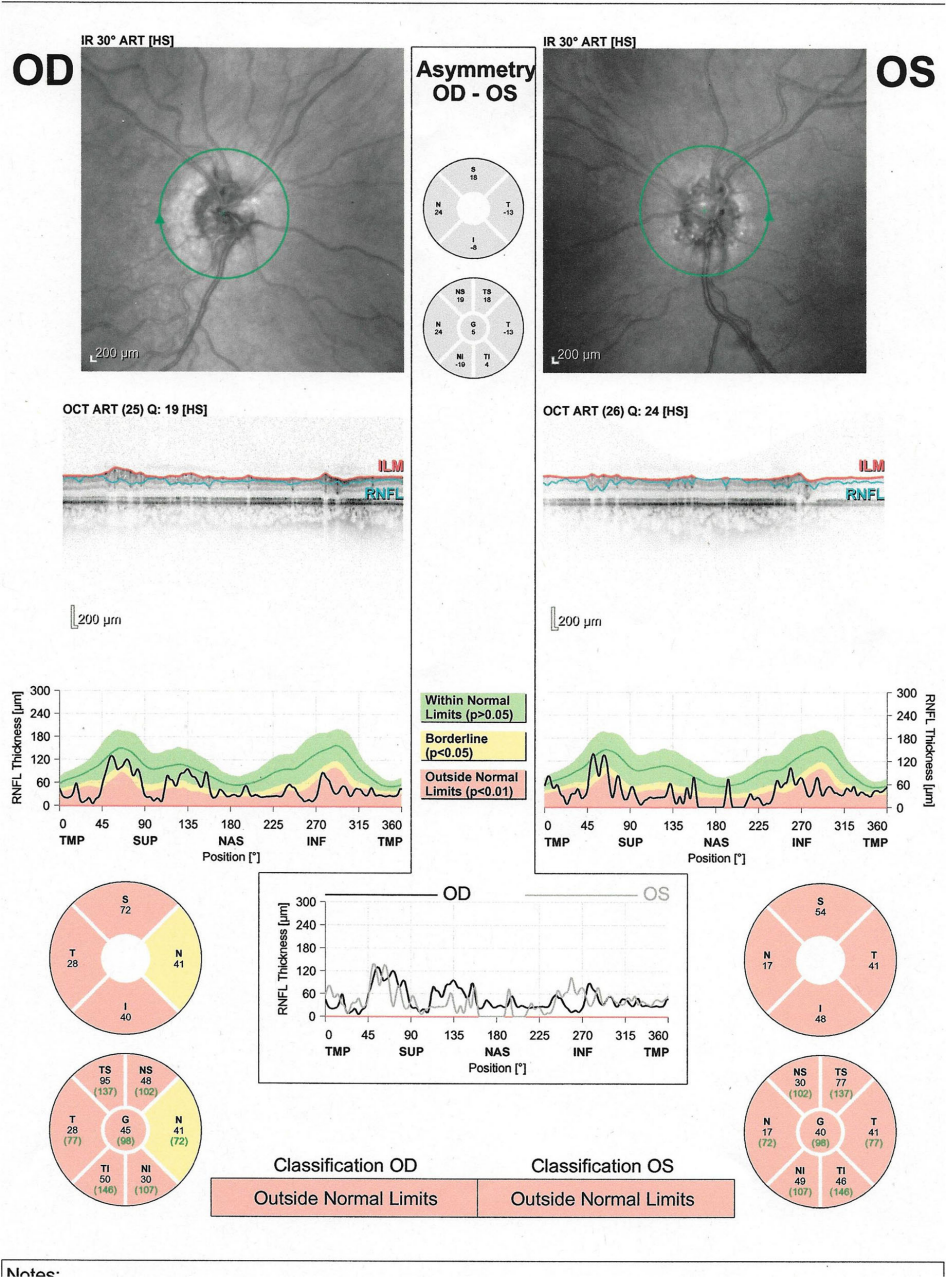
OCT of the optic nerve: widespread loss of optic nerve fibers in both eyes

This was confirmed by optical coherence tomography (OCT HRA-II, Heidelberg Engineering, Heidelberg, Germany), to further examine the optic nerve. The fast optic disk scan protocol showed shadowing of the deep optic nerve consistent with ONHD. The fast retinal nerve fiber layer thickness scan and analysis indicated advanced thinning in each eye, which helps depict the general health of the optic nerve (Fig. 7). The advanced nerve fiber thinning supported the degree of field loss found on visual field testing.

We checked her parents’ eyes in January 2015. They were both pseudophakic; the mother and father had undergone surgery for cataract extraction in OU at the ages of 60 and 62 years, respectively. They had no changes in the anterior and posterior segments and intraocular pressure and orthoptic findings were normal.

To search for other possible genetic causes of this complex clinical presentation, we applied NGS analysis on DNA of the proband and her parents. Library preparation and target selection were done using Illumina Nextera Coding Exome with subsequent sequencing on an Illumina HiSeq sequencer, in 2 × 100 sequencing mode. Reads were aligned to UCSC hg19 reference assembly using the BWA algorithm (v 0.6.3) and variant calling was done with the GATK framework (v 2.8). Only variants exceeding the quality score of 30.0 and depth of 5 were used for downstream analyses. Variant annotation was done with ANNOVAR and snp Eff algorithms, with pathogenicity predictions in the dbNSFPv2 database. Reference gene models and transcript sequences are based on the RefSeq database. Structural variants were assessed using the CONIFER v0.2.2 algorithm. Variants were evaluated according Meynert AM et al. [2].

Variants for the case were filtered according to de novo, autosomal recessive and X-linked models of inheritance. Taking into account the severity and penetrance of the sought genetic variation, we filtered out population variants attaining frequency above 0.01% in any of the surveyed populations for de novo model and filtered out variants exceeding 0.1% in the autosomal recessive model. All suspect de novo variants were also visually inspected at aligned read level with the aim of avoiding false call due to misalignment or low-depth of coverage in parental samples. Synonymous variants and intronic variants more than 20 base pairs away form the intron-exon junctions were disregarded from further interpretation steps. The genes analyzed are listed in Table 2.

**Table 2:** Lists of genes analyzed in focused exome data analyses. The HPO panels were generated using the gene-phenotype associations in Human Phenotype Ontology project data. Other panels were constructed based on the existing gene-disease associations

Virtual gene panel	List of genes in the panel
1 gene associated with optic nerve drusen presentation	MFRP
132 genes, associated with a broad set of genes associated with cataract phenotype (HP:0000518)	ABCB6, ADAMTS10, ADAMTSL4, AGK, AGPS, AKR1E2, ALDH18A1, B3GALTL, BCOR, BFSP1, BFSP2, CBS, CHMP4B, COL11A1, COL18A1, COL2A1, COL4A1, COL4A2, CRYAA, CRYAB, CRYBA1, CRYBA4, CRYBB1, CRYBB2, CRYBB3, CRYGB, CRYGC, CRYGD, CRYGS, CTDP1, CUTL1, CYP27A1, CYP51A1, DHCR7, EPG5, EPHA2, ERCC1, ERCC2, ERCC3, ERCC5, ERCC6, ERCC8, EYA1, FAM126A, FBN1, FKRP, FKTN, FOXC1, FOXD3, FOXE3, FTL, FYCO1, FZD4, GALK1, GALT, GCNT2, GJA1, GJA3, GJA8, GLA, GNPAT, HMX1, HSF4, JAM3, L1CAM, LARGE, LEPREL1, LIM2, LMX1B, LRP5, LTBP2, MAF, MAN2B1, MFSD6L, MIP, MIR184, MYH9, NDP, NF2, NHS, OCRL, OPA3, PAX6, PEX1, PEX10, PEX11β, PEX12, PEX13, PEX14, PEX16, PEX19, PEX2, PEX26, PEX3, PEX5L, PEX6, PEX7, PITX2, PITX3, POMT1, POMT2, PVRL3, PXDN, RAB18, RAB3GAP1, RAB3GAP2, RECQL2, RECQL4, RNLS, SC5DL, SEC23A, SIL1, SIX5, SIX6, SLC16A12, SLC2A1, SLC33A1, SOLH, SORD, SOX2, SRD5A3, SREBF2, TBC1D20, TDRD7, TFAP2A, TMEM114, TMEM70, VAV2, VAV3, VIM, VSX2, WFS1
255 genes in an expanded eye panel	ABCA4, ABHD12, ACAD11, ADAM9, AHI1, AIPL1, ALMS1, ALX3, ALX4, ANK2, ANKRD1, ANO5, AP3B1, ARL13B, ARL6, BBS1, BBS10, BBS12, BBS2, BBS4, BBS5, BBS7, BBS9, BCOR, BEST1, BLOC1S3, BLOC1S6, BMP4, C1QTNF5, C2orf71, C2ORF71, C5orf42, C8orf37, C8ORF37, CA4, CABP4, CACNA1F, CACNA2D4, CAPN5, CC2D2A, CCDC28B, CDH23, CDH3, CDHR1, CEP164, CEP290, CEP41, CERKL, CHM, CIB2, CLN3, CLRN1, CNGA1, CNGA3, CNGB1, CNGB3, CNNM4, COL11A1, COL11A2, COL2A1, COL9A1, COL9A2, CRB1, CRX, CRYBA4, CYP1B1, CYP4V2, DFNB31, DHDDS, DHODH, DTNBP1, EFEMP1, EFNB1, ELOVL4, EVC, EVC2, EYS, FAM161A, FKRP, FKTN, FLVCR1, FOXC1, FOXE3, FRAS1, FREM1, FREM2, FSCN2, FZD4, GNAT1, GNAT2, GNPTG, GPR143, GPR179, GPR98, GRK1, GRM6, GUCA1A, GUCA1B, GUCY2D, HARS, HCCS, HMCN1, HPS1, HPS3, HPS4, HPS5, HPS6, IDH3B, IL11RA, IMPDH1, IMPG2, INPP5E, INVS, IQCB1, JAG1, KCNJ13, KCNV2, KIF7, KLHL7, LARGE, LCA5, LRAT, LRIT3, LRP5, LYST, LZTFL1, MAK, MC1R, MERTK, MFN2, MFRP, MKKS, MKS1, MSX2, MTTP, MYO7A, MYOC, NDP, NMNAT1, NPHP1, NPHP3, NPHP4, NR2E3, NRL, NYX, OAT, OCA2, OFD1, OPA1, OPA3, OPTN, OTX2, PANK2, PAX2, PAX6, PCDH15, PDE6A, PDE6B, PDE6C, PDE6G, PDE6H, PDZD7, PEX1, PEX2, PEX26, PEX7, PHYH, PITPNM3, PITX2, PITX3, PLA2G5, POLR1C, POMGNT1, POMT1, POMT2, PRCD, PROM1, PRPF3, PRPF31, PRPF6, PRPF8, PRPH2, RAB28, RAX2, RB1, RBP3, RBP4, RD3, RDH12, RDH5, RECQL4, RGR, RGS9, RGS9BP, RHO, RIMS1, RLBP1, ROM1, RP1, RP1L1, RP2, RP9, RPE65, RPGR, RPGRIP1, RPGRIP1L, RS1, SAG, SDCCAG8, SEMA4A, SIX6, SLC24A1, SLC45A2, SMOC1, SNRNP200, SOX2, SPATA7, SPG7, STRA6, TCOF1, TCTN1, TCTN2, TCTN3, TIMM8A, TIMP3, TMEM126A, TMEM138, TMEM216, TMEM231, TMEM237, TMEM67, TOPORS, TREX1, TRIM32, TRPM1, TSPAN12, TTC21B, TTC8, TTPA, TULP1, TWIST1, TYR, TYRP1, UNC119, USH1C, USH1G, USH2A, VAX1, VCAN, VPS13B, VSX2, WDPCP, WFS1, ZNF423, ZNF513

We have utilized two approaches to collect the genes - we included genes that are associated with Cataract (HP:0000518) phenotypes, utilizing data collected within Human Phenotype Ontology database [3]. Additionally, we supplanted the set of genes with the genes, associated with cataract in the database of panels, collected in the EuroGenTest NGS panels database [4].

For DGS the proband’s DNA was analyzed using the SurePrint G3 human microarray-CGH kit 8x60K (Agilent). Detailed methods for genomic DNA preparation, labeling, hybridization and scanning can be found at websitehttp://www.home.agilent.com.

We identified compound heterozygosity for two known pathogenic variants associated with GSD Ia (OMIM:232,200). Whole human exome sequencing did not show any genetic variants that could adequately explain the patient’s ocular presentation, focusing on the genes associated with ocular phenotypes. We did not identify any plausible candidates gene variants adhering to the recessive and X-linked models of inheritance. We did identify one de novo missense variant of unknown significance in the USP13 gene (NM_003940.2:c.1544 T > C, p.Ile515Thr) with conflicting theoretical predictions of pathogenicity. Furthermore, the USP13 gene itself has not been associated with the cataract phenotype in the patient in clinical or functional studies and there is currently no evidence to support the role of this variant in the patient’s disease.

However, the analysis used cannot exclude the presence of pathogenic intronic variants, pathogenic deletions or duplications, trinucleotide expansions and pathogenic variants in gene regions that are not captured. Also, it was not possible to exclude pathogenic variants in genes not included in the clinical target.

Our results confirmed the previously identified genetic defects responsible for GSD and DGS, but no specific genetic causes were found for the cataract and/or ONHD.

## Discussion

No clear association between GSD Ia and ocular findings has been reported so far, probably because studies included only small groups of patients. Fine et al. described multiple, bilateral, symmetric, yellowish, non-elevated, discrete paramacular retinal lesions in patients with GSD Ia, very likely due to specific lipid or glycogen deposition [5]. Allegrini et al. described multiple, bilateral, punctate and peripheral opacities of the lens in 11 patients who had had GSD Ia or III for more than 20 years [6].

The complex case described here presented some typical eye signs of DGS, such as retinal vascular tortuosity, eyelid hooding, strabismus and astigmatism [7]. Hypocalcemia, which may be present in DGS, is a known cause of cataract in humans [8]; however, there are no published reports of cataract associated with DGS. The influence of a calcium-free environment on membrane permeability of the lens has been extensively examined [9–11]. In vitro studies have been concerned primarily with the short-term effect on the lens of calcium levels so low as to be unphysiological. Clinically, it is recognized that lens changes associated with hypocalcemia develop over a variable timespan. In vivo, during the early stages of hypocalcemia, the lens may employ compensating mechanisms to overcome small permeability changes. Delamere et al. described lens changes in hypocalcemic rabbits; the lenses developed posterior subcapsular punctate opacities with no cortical involvement, but affecting most of the posterior subcapsular region with time [12]. The pattern of development of lens opacities was similar to that described in our case and in human studies6. Lens changes in the patient described here were: a) subcapsular and b) multiple, punctate and peripheral opacities; this is in agreement with other published findings [6, 8–12].

The etiology of ONHD is still not clear. Many studies support Lorentzen’s theory of an irregularly dominant inheritance pattern resulting in approximately ten times the prevalence rate found in the general population [13–16]. An investigation in 1999 concluded that the primary pathology of optic disk drusen is most likely an inherited dysplasia of the optic disk and its blood supply, which would predispose to drusen formation [17]. An alternative explanation for the inheritance of optic disk drusen could be inheritance of the optic disk size, which by itself is a risk factor for the development of ONHD [18]. Although scleral canal size is not an etiologic factor in the pathogenesis of ONHD, the canal can be measured using OCT in patients with optic nerve drusen [19]. A congenitally abnormal disk vasculature may allow transudation of plasma proteins which can then serve as a nidus for the deposition of extracellular materials, facilitating the progression of drusen [20].

Although the presence of cataract and ONHD could be a coincidence; the case reported here might suggest that they could have a common pathogenesis. In fact Ringvold et al. suggest that hypocalcemic cataracts contain increased amounts of calcium; this is the first report of such a finding in human specimens [21], and it agrees with similar experimental observations from different species [22–25]. It also fits with the observation that hypocalcemic patients may acquire calcium deposits in the brain and other organs [26]; this theory could explain the drusen in our case. ONHD contain mucopolysaccharides, aminoacids, ribonucleic and desoxyribonucleic acid, with small amounts of iron and especially calcium [27–29].

## Conclusions

The association between GSD I and cataract is known in literature [6], instead it has not been previously reported cases of GSD I and DGS associated with ONHD.

There are several pathogenic hypotheses concerning both the onset of cataracts in metabolic syndromes and the formation of ONHD. Our genetic study on the patient and her parents had the objective of verifying whether cataract and/or ONHD had genetic causes, but no specific causative mutation was found. However, the etiology of the lens changes observed are in agreement with the literature [6, 8–12], where the pathogenesis of ONHD is still not clear.

Although in the case reported the presence of ONHD could be a coincidence, it will be interesting in the future: a) to assess their incidence in patients suffering from DGS and hypocalcemic diseases to check whether they can be caused by metabolic disorders; b) to do NGS studies on patients with ONHD and their parents to examine whether the drusen could be caused by mutations of recessive genes not yet known.

## Abbreviations

DGS: DiGeorge syndrome
G6P: Glucose-6-phosphatase
GSD: With glycogenosis
OD: Right eye
ONHD: Optic nerve head drusen
OS: Left eye
OU: Both eyes
